# Supplementary material for: Construction and Validation of a Brief Pandemic Fatigue Scale in the Context of the Coronavirus-19 Public Health Crisis
Source: Int J Public Health. 2021 Aug 30;66:1604260. doi: 10.3389/ijph.2021.1604260 (PMC8461461; doi:10.3389/ijph.2021.1604260)
Supplement: Supplementary file 2 [file DataSheet1.zip › SupplementaryFigure1.docx]

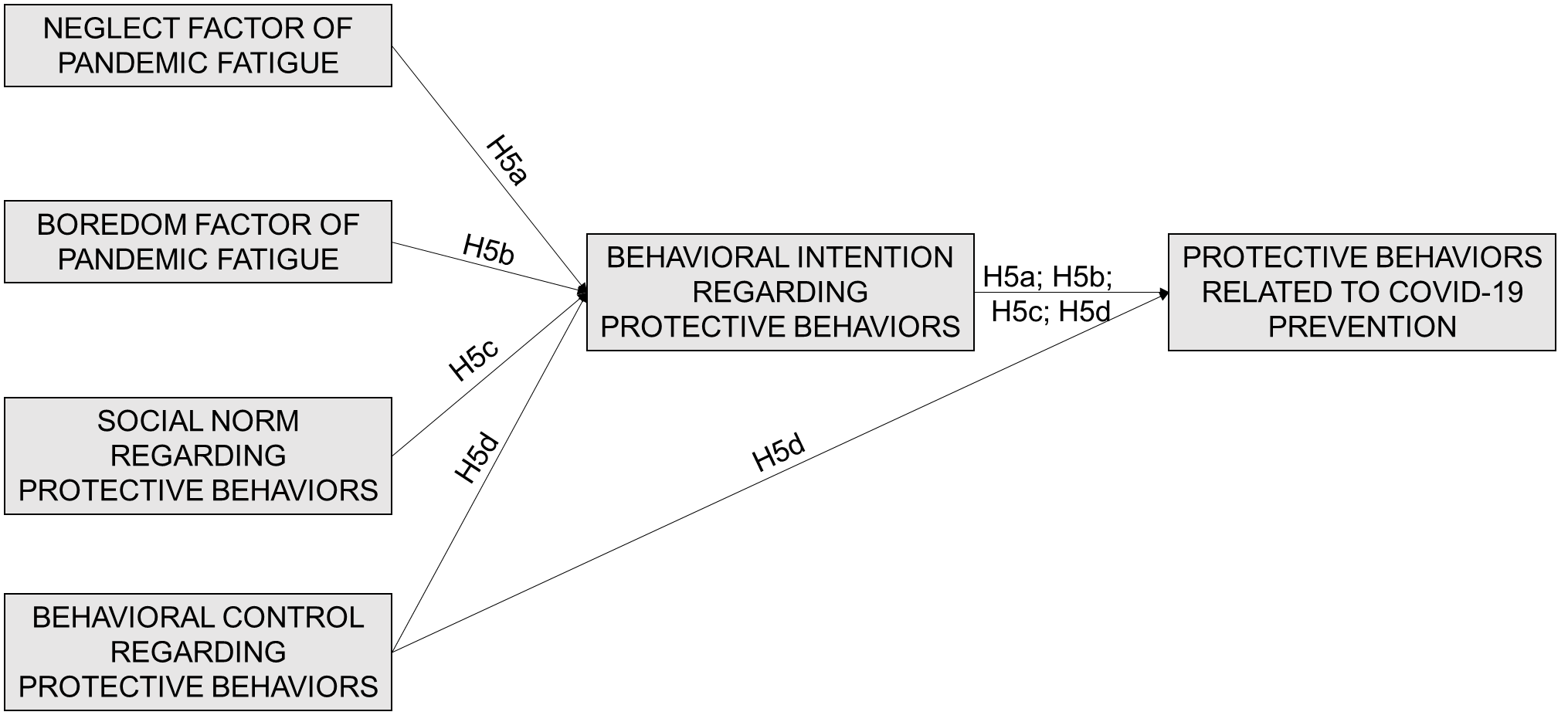
 **Supplementary Figure 1.** *Predictive Model of Protective Behaviors Related to Coronavirus-19 Prevention with Pandemic Fatigue as Predictive Determinant and on the basis of the Theory of Planned Behavior* (11,12)*. Adapted from Madden et al.* (13)
