# Supplementary material for: Construction and Validation of a Brief Pandemic Fatigue Scale in the Context of the Coronavirus-19 Public Health Crisis
Source: Int J Public Health. 2021 Aug 30;66:1604260. doi: 10.3389/ijph.2021.1604260 (PMC8461461; doi:10.3389/ijph.2021.1604260)
Supplement: Supplementary file 2 [file DataSheet1.zip › SupplementaryTable1.docx]

| **Supplementary Table 1**. *Sociodemographic Characteristics of the Participants* | | | | | | | |
| --- | --- | --- | --- | --- | --- | --- | --- |
|  | Pilot sample | | |  | Global Sample | | |
|  | *N* | Valid % | *M* (*sd)* |  | *N* | Valid % | *M* (*sd)* |
| Gender | | | | | | | |
| Women | 226 | 75.3 | - |  | 384 | 64.4 | - |
| Men | 74 | 24.7 | - |  | 212 | 35.6 | - |
| Age | - | - | 19.21  (1.65) |  | - | - | 42.62  (13.90) |
| Place of residence | | | | | | | |
| Andalusia | 248 | 82.7 | - |  | 439 | 73.7 | - |
| Basque Country | 1 | .3 | - |  | 11 | 1.8 | - |
| Castille and Leon | 2 | .7 | - |  | 11 | 1.8 | - |
| Castille La Mancha | 11 | 3.7 | - |  | 15 | 2.5 | - |
| Catalunya | 4 | 1.3 | - |  | 15 | 2.5 | - |
| Extremadura | 5 | 1.7 | - |  | 5 | .8 | - |
| Madrid | 7 | 2.3 | - |  | 28 | 4.7 | - |
| Elsewhere in Spain | 7 | 2.3 | - |  | 43 | 7.2 | - |
| Other countries | 6 | 1.8 | - |  | 16 | 3.1 | - |
| Job situation |  |  |  |  |  |  |  |
| Worker or student | 299 | 99.7 | - |  | 473 | 79.4 | - |
| *Employed worker* | 10 | 3.3 | - |  | 374 | 62.8 | - |
| *Student* | 289 | 96.3 | - |  | 99 | 16.6 | - |
| Jobless | 0 | 0 | - |  | 97 | 16.2 | - |
| *Unemployed* | 0 | 0 | - |  | 55 | 9.2 | - |
| *Retired* | 0 | 0 | - |  | 42 | 7 | - |
| *Other* | 1 | .3 | - |  | 26 | 4.4 | - |
| Reported not living with someone who has an illness or chronic health problem that makes them especially vulnerable to Covid-19 | 156 | 52 | - |  | 361 | 60.6 | - |
|  |  |  |  |  |  |  |  |
| Reported not having contracted Covid-19 | 274 | 91.3 | - |  | 563 | 94.5 | - |
|  |  |  |  |  |  |  |  |
| Reported having no family member or close friend who had contracted Covid-19 | 244 | 81.3 | - |  | 471 | 79 | - |
|  |  |  |  |  |  |  |  |
| Reported that a family member or close friend had died from Covid-19 | 53 | 17.7 | - |  | 168 | 28.2 | - |
|  |  |  |  |  |  |  |  |
