# Supplementary material for: Construction and Validation of a Brief Pandemic Fatigue Scale in the Context of the Coronavirus-19 Public Health Crisis
Source: Int J Public Health. 2021 Aug 30;66:1604260. doi: 10.3389/ijph.2021.1604260 (PMC8461461; doi:10.3389/ijph.2021.1604260)
Supplement: Supplementary file 2 [file DataSheet1.zip › SupplementaryTable2abcd.docx]

| **Supplementary Table 2.** *Ad-hoc scales* | | | | | | | |
| --- | --- | --- | --- | --- | --- | --- | --- |
| **Supplementary Table 2a. Perceived Social Norm Scale** | | | | | | | |
| Here are some sentences that describe how the people with whom you usually interact think about and behave in relation to Coronavirus-19. Please indicate if you agree with those sentences by using the proposed scale (1 = Absolutely disagree; 7 = Absolutely agree). | | | | | | | |
| ***The people who I usually interact with...*** | | | | | | | |
| In general, they believe that wearing the mask everywhere is exaggerated | 1 | 2 | 3 | 4 | 5 | 6 | 7 |
| In general, they believe that to ventilate indoor spaces continuously is exaggerated | 1 | 2 | 3 | 4 | 5 | 6 | 7 |
| In general, they believe that it is not a problem to meet many people indoors | 1 | 2 | 3 | 4 | 5 | 6 | 7 |
| In general, they believe that it is exaggerated not to meet for festivities in order to avoid the spread of Covid | 1 | 2 | 3 | 4 | 5 | 6 | 7 |
| In general, they believe that the set of measures that are said to be adopted are exaggerated | 1 | 2 | 3 | 4 | 5 | 6 | 7 |
| In general, they believe that not to be able to meet in pubs/restaurants is exaggerated | 1 | 2 | 3 | 4 | 5 | 6 | 7 |
| They take off their masks when they are with friends indoors | 1 | 2 | 3 | 4 | 5 | 6 | 7 |
| They open the windows when they are with people not living together indoors* | 1 | 2 | 3 | 4 | 5 | 6 | 7 |
| They meet with other people indoors (pubs, restaurants, homes, etc.) | 1 | 2 | 3 | 4 | 5 | 6 | 7 |
| They keep their distance from other people with whom they do not live* | 1 | 2 | 3 | 4 | 5 | 6 | 7 |
| They frequently wash/disinfect their hands* | 1 | 2 | 3 | 4 | 5 | 6 | 7 |
| **Supplementary Table 2b. Perceived Behavioral Control Scale** | | | | | | | |
| Please indicate to what extent you think the following prevention measures are easy to follow by using the proposed scale (1 = Very difficult; 7 = very easy). | | | | | | | |
| ***Do you think it's easy to carry out the following measures?*** | | | | | | | |
| To avoid meeting indoors with family and friends who are not living together | 1 | 2 | 3 | 4 | 5 | 6 | 7 |
| To meet family and friends who are not living together only outdoors | 1 | 2 | 3 | 4 | 5 | 6 | 7 |
| To maintain a security distance of at least 6 feet from my family and friends with whom I am not usually living | 1 | 2 | 3 | 4 | 5 | 6 | 7 |
| To open the windows continuously in my house when there is a person with whom I do not usually live, even if it is only a few centimeters | 1 | 2 | 3 | 4 | 5 | 6 | 7 |
| To filter the air in my home when there are people with whom I do not live | 1 | 2 | 3 | 4 | 5 | 6 | 7 |
| To wear a mask all the time during a family reunion with people who do not live with me, except when we eat or drink | 1 | 2 | 3 | 4 | 5 | 6 | 7 |
| To be tested to know if I am contagious before meeting with people I do not live with | 1 | 2 | 3 | 4 | 5 | 6 | 7 |
| To wash/disinfect my hands frequently | 1 | 2 | 3 | 4 | 5 | 6 | 7 |
| To clean surfaces frequently | 1 | 2 | 3 | 4 | 5 | 6 | 7 |
| **Supplementary Table 2c. Protective Behavioral Intention Scale** | | | | | | | |
| Here are some sentences that represent some behaviors that you can do during family gatherings in the next Christmas festivities with people with whom you do not live habitually. Please indicate to what extent you have the intention to do these behaviors. (1 = Never; 7 = Always) | | | | | | | |
| ***At our family gatherings (lunches and dinners) during the festivities...*** | | | | | | | |
| We will take off the mask only when we are eating or drinking | 1 | 2 | 3 | 4 | 5 | 6 | 7 |
| We will keep the windows open during all the time we are together | 1 | 2 | 3 | 4 | 5 | 6 | 7 |
| We will filter the air with a mechanical filtration device (purifiers, etc.) | 1 | 2 | 3 | 4 | 5 | 6 | 7 |
| We will keep a social distance of at least 6 feet while we do not wear a mask | 1 | 2 | 3 | 4 | 5 | 6 | 7 |
| We will do be tested before each family meal/dinner, and if one of us is positive, we will not meet with him/her or his/her usual living-together group | 1 | 2 | 3 | 4 | 5 | 6 | 7 |
| As much as possible, we will self-confine in the days before family gatherings | 1 | 2 | 3 | 4 | 5 | 6 | 7 |
| We will meet without adopting any special measures, as we do every Christmas* | 1 | 2 | 3 | 4 | 5 | 6 | 7 |
| We will not wear masks while we are gathering* | 1 | 2 | 3 | 4 | 5 | 6 | 7 |
| **Supplementary Table 2d. Protective Behavior Scale** | | | | | | | |
| Here are some protective behaviors that individuals can do to avoid catching Covid. Please indicate to what extent you adopt those behaviors by using the proposed scale (1 = Never; 7 = Always) | | | | | | | |
| ***How often (from 1 to 7) do you adopt the protective measures proposed to avoid contracting Covid?*** | | | | | | | |
| I wash my hands frequently | 1 | 2 | 3 | 4 | 5 | 6 | 7 |
| Indoors, I always wear a mask when I am with people I do not live with (at the office, at home with non-living-together family or friends, etc.) | 1 | 2 | 3 | 4 | 5 | 6 | 7 |
| Before leaving, I take the time to adjust my mask correctly, making sure that there are not any gaps through which air can pass | 1 | 2 | 3 | 4 | 5 | 6 | 7 |
| I avoid touching my face (eyes, mouth, and nose) | 1 | 2 | 3 | 4 | 5 | 6 | 7 |
| Indoors, if I am with people I do not live with, I leave the windows open all the time, even if it is not wide open | 1 | 2 | 3 | 4 | 5 | 6 | 7 |
| I avoid meeting my friends and family members who are not living together indoors | 1 | 2 | 3 | 4 | 5 | 6 | 7 |
| I avoid crowded places and events | 1 | 2 | 3 | 4 | 5 | 6 | 7 |
| I spend more time at home | 1 | 2 | 3 | 4 | 5 | 6 | 7 |
| I keep a secure distance from others | 1 | 2 | 3 | 4 | 5 | 6 | 7 |

* Reversed items
