# Supplementary material for: Construction and Validation of a Brief Pandemic Fatigue Scale in the Context of the Coronavirus-19 Public Health Crisis
Source: Int J Public Health. 2021 Aug 30;66:1604260. doi: 10.3389/ijph.2021.1604260 (PMC8461461; doi:10.3389/ijph.2021.1604260)
Supplement: Supplementary file 2 [file DataSheet1.zip › SupplementaryTable3.docx]

| **Supplementary Table 3.** *Correlation Between the Global* *PFS, the Neglect Factor, and the Boredom Factor with the Different Study Variables* | | | | | |
| --- | --- | --- | --- | --- | --- |
|  | Pandemic Fatigue Scale | Neglect factor | Boredom Factor | Mean | sd |
| Pandemic Fatigue Scale | - | . |  | 2.98 | 1.47 |
| Neglect factor | .84*** | - |  | 2.25 | 1.65 |
| Boredom factor | .87*** | .46*** | - | 3.71 | 1.79 |
| Security Social Value Orientation | -.14*** | -.17*** | -.08^#^ | 5.61 | 1.35 |
| Conformism Social Value Orientation | -.09* | -.12** | -.04 | 5.56 | 1.37 |
| Social norm perception | .19*** | .24*** | .08* | 2.75 | 1.45 |
| Perceived behavioral control | -.18*** | -.25*** | -.06 | 4.80 | 1.38 |
| Protective behavior intention | -.34*** | -.32*** | -.28*** | 4.05 | 1.38 |
| Protective behavior | -.14*** | -.20*** | -.05^#^ | 5.64 | 1.44 |
| ^#^*p* < .09. **p* < .05. ***p* < .01 ****p* < .001. | | | | | |
